# Current advances and outlooks in immunotherapy for pancreatic ductal adenocarcinoma

**DOI:** 10.1186/s12943-020-01151-3

**Published:** 2020-02-15

**Authors:** Jia-qiao Fan, Meng-Fei Wang, Hai-Long Chen, Dong Shang, Jugal K. Das, Jianxun Song

**Affiliations:** 1 Third General Surgery Department, The First Affiliated Hospital of Dalian Medical University, Dalian, China; 2 Department of Microbial Pathogenesis and Immunology, Texas A&M University Health Science Center, College Station, TX USA

**Keywords:** Pancreatic ductal adenocarcinoma, Immunotherapy, Tumour microenvironment, Myeloid-derived suppressor cells, Regulatory T lymphocytes, Tumour-associated macrophages, Tumour-associated antigens, Adoptive cell therapy, Immune checkpoint inhibitor, Tumour-infiltrating lymphocytes, Vaccines, Neoantigens

## Abstract

Pancreatic ductal adenocarcinoma (PDAC) is an incurable cancer resistant to traditional treatments, although a limited number of early-stage patients can undergo radical resection. Immunotherapies for the treatment of haematological malignancies as well as solid tumours have been substantially improved over the past decades, and impressive results have been obtained in recent preclinical and clinical trials. However, PDAC is likely the exception because of its unique tumour microenvironment (TME). In this review, we summarize the characteristics of the PDAC TME and focus on the network of various tumour-infiltrating immune cells, outlining the current advances in PDAC immunotherapy and addressing the effect of the PDAC TME on immunotherapy. This review further explores the combinations of different therapies used to enhance antitumour efficacy or reverse immunodeficiencies and describes optimizable immunotherapeutic strategies for PDAC. The concordant combination of various treatments, such as targeting cancer cells and the stroma, reversing suppressive immune reactions and enhancing antitumour reactivity, may be the most promising approach for the treatment of PDAC. Traditional treatments, especially chemotherapy, may also be optimized for individual patients to remodel the immunosuppressive microenvironment for enhanced therapy.

## Introduction

PDAC remains one of the deadliest malignancies with a poor outcome, and very few regimens have been successfully used to treat this lethal cancer. The 5-year overall survival (OS) rate of PDAC patients is abysmal at less than 5% [1]. PDAC was the fourth leading cause of cancer-related death in 2012 [2] and is projected to become the third most common cancer in the United States by 2030. Although PDAC-associated morbidity does not rank highly in cancer epidemiology [3], the mortality rate is nearly the highest among of all cancers. Surgical resection is the sole curable approach for localized PDAC, but no more than 20% of tumours are resectable at the time of diagnosis due to the lack of early symptoms and the aggressive biological nature of this carcinoma [4]. Most patients relapse after surgery even after routine adjuvant therapies have been used systematically [5]. Neoadjuvant treatment increases the resectable rate and benefits OS, but the results are unclear [6]. Even for patients with localized and resectable tumours, the 5-year OS rate is only approximately 27% [7]. Chemotherapy based on gemcitabine (Gem) is currently the standard treatment for metastatic PDAC, and the combination of Gem with oxaliplatin, irinotecan, leucovorin and 5-fluorouracil (FOLFIRINOX) can reduce the mortality rate but has been shown to increase toxicity and to have a poor survival benefit and high cost burden [8, 9]. Therefore, the exploration of new therapies for PDAC is urgently needed. Immunotherapy, including strategies such as monoclonal antibody (mAb) therapy, immune checkpoint inhibitor (ICI) therapy, adoptive cell therapy/adoptive cell transfer (ACT), vaccines and other agents that enhance the antitumour response and/or reverse the immunosuppressive functions of regulatory immune cells in the TME, has made great progress in cancer treatment in recent decades. However, no immunotherapeutic approaches have produced promising results thus far despite similar strategies making notable progress in other cancers. For reasons unknown, the TME plays a critical role in the development, progression, and metastasis of PDAC as well as to its sensitivity to immunotherapy.

## TME of PDAC

**Fig. 1:**
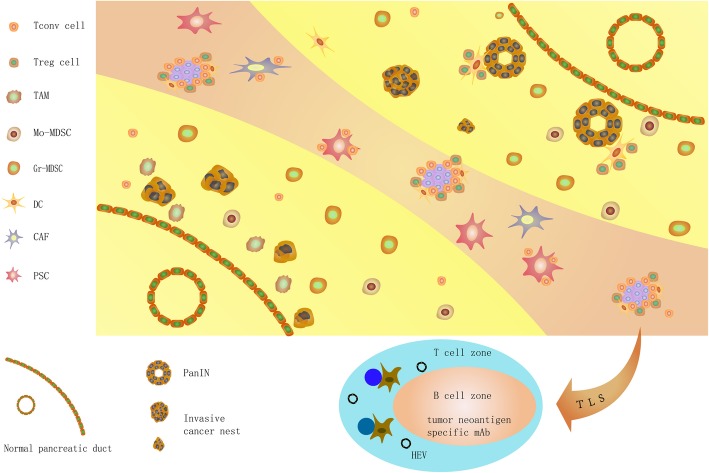
The graphic abstract of PDAC TME.• From the right upper side to the left low side, we summarize the progression of PDAC from PanIN and the distribution of different cells in TME. The yellow area represent the area mainly comprising different advanced stage of epithelial tissue from normal acinar to PanIN and invasive cancer nest, as well as monocyte-like cells; the reddish area present the area comprising mainly matrix including fibrotic matrix, pancreatic stellate cells, cancer associated fibroblasts, TLS, as well as accumulated effector lymphocytes. The cancer nests look like islands in the stroma desert; Treg cells surround the PanIN and establish a TSA specific suppressive condition to support PDAC progression; MDSCs appear at very early stage of the PDAC progression and disperse the whole lesion of tumor; TAMs locate majorly at the invasive front of the tumor and promote angiogenesis, lymphogenesis and metastasis; DCs are scarce and restricted in PanIN and TLS; CAFs and PSCs are the major source of tumor stromal matrix, they can also adhere infiltrating T lymphocytes, keep them outside of cancer nest and result effector T cell anergy; TLS localize in the tumor stroma and consist of proliferating effector cells as well as Treg cells, tumor specific anti-tumor and pro-tumor reactivity present concordantly

**Fig. 2:**
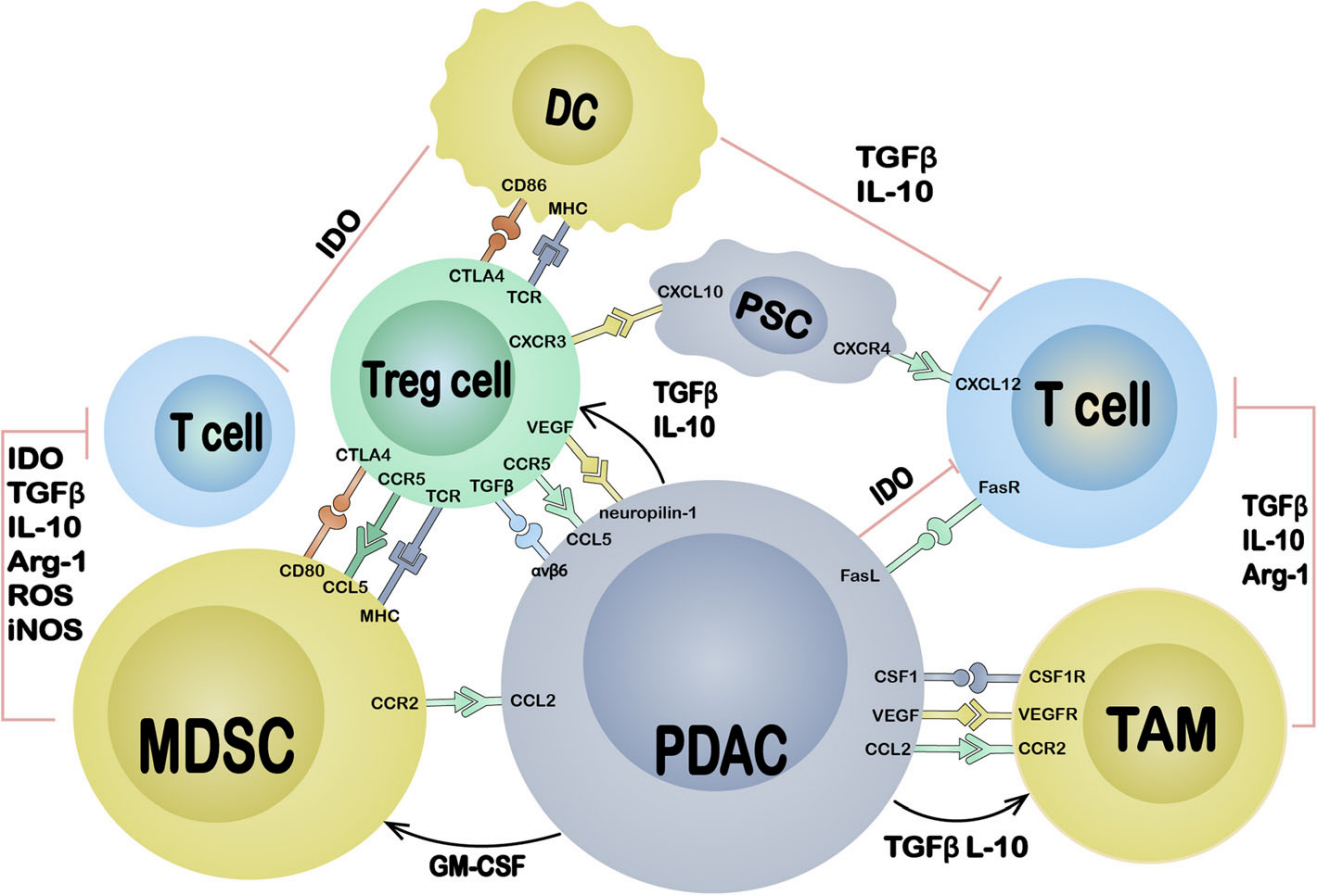
The molecular interaction of different cells in TME. The cancer cells of PDAC exploit several mechanisms including cell surface molecule and soluble factors to establish immunosuppressive TME through accumulating and activating immune suppressive cells, and inhibiting antitumor effector cells directly and indirectly; suppressive cells can inhibit the function of effector cells through nutrition depletion, phenotype alternation, apoptosis and anergy; Treg cells may play a central role in the establishment of immunosuppressive TME of PDAC since they are in favor of establishing tumor specific immunotolerance and have extensive interaction with other cells

The TME of PDAC consists of the cancer cell nest and stroma. The stroma contains various components, primarily the stromal matrix and various cells. Here, we concisely summarize the existing knowledge about the TME of PDAC (Fig. 1) and emphasize the immune cell network established around cancer cells (Fig. 2).

### PDAC epithelial cells

Tumour-associated antigens (TAAs) have been identified in PDAC but are limited, and the absence of TAAs hinders naturally occurring antitumour reactivity. Deficiencies in antigen processing and epitope presentation are another critical mechanism of immune evasion. PDAC cells generally downregulate the expression of major histocompatibility (MHC) class I molecules [10–12], and MHC class I/II molecules may also develop genetic mutations that result in impaired antigen presentation. Aberrant expression of the receptor Fas and Fas ligand extensively occurs in most PDAC patients and results in immune tolerance. Normal pancreatic ductal cells express the Fas receptor but not the Fas ligand, while PDAC cells express a non-functional form of the Fas receptor, which results in resistance to Fas-mediated apoptosis; furthermore, PDAC cells express the Fas ligand to induce apoptosis in immune effector cells [13]. PDAC cells recruit immunosuppressive tumour-associated macrophages (TAMs) and myeloid-derived suppressor cells (MDSCs) from the peripheral circulation via the CCL2/CCR2 axis [14]. PDAC cells express high levels of CCL5 to recruit regulatory T cells (Treg cells) through CCR5 [15], and this process may partially explain the recruitment of Treg cells to PDAC lesions [16]. Approximately 12.5% of PDAC patients are reported to positively express programmed cell death protein ligand-1 (PD-L1) [17], which induces T cell anergy and apoptosis through programmed cell death protein-1 (PD-1) expressed on T cells, resulting in immune system evasion [18]. PDAC cells can also programme the TME by directly secreting soluble cytokines, such as transforming growth factor (TGF-β) and interleukin (IL)-10, to inhibit dendritic cell (DC) differentiation and maturation in favour of Treg cell accumulation [19, 20]. PDAC cells produce indoleamine 2,3-dioxygenase (IDO) to catalyse the degradation of tryptophan, which is necessary for T cell survival and activation, thereby inducing T cell apoptosis and anergy [21, 22].

### PDAC stroma

A high-density fibrotic stromal reaction, termed “desmoplasia”, may be one of the most prominent characteristics of the PDAC stroma, as almost 90% of the tumour mass is composed of the stroma, which facilitates immunosuppression and fibrosis progression [23, 24]. The carcinogenic nests appear as islands surrounded by the stromal desert, as depicted in Fig. 1. The PDAC stroma has been demonstrated to not only promote tumour progression but to also dampen the delivery of antitumour regimens [24–26], even increasing the number of immunosuppressive cells and inactivating cytotoxic CD8^+^ T cells [27, 28]. Controversial results have been reported recently, including those of Wang and Knudsen et al., who divided PDAC into three classes according to the stromal density and demonstrated that stromal density and volume had a positive association with patient OS [29, 30]. Özdemir et al. interpreted the mechanisms in a preclinical study in which cancer-associated fibroblasts (CAFs) were depleted, which had extensive effects on the TME, such as reducing collagen and matrix reorganization, decreasing angiogenesis, enhancing hypoxia, increasing cancer stem cell numbers, and increasing Treg cell frequency, all of which contributed to a poor outcome [31]. The numbers of pancreatic stellate cells (PSCs), special CAFs unique to PDAC, increase abundantly during the progression of the disease [32]. Activated PSCs can restrain tumour-infiltrating CD8^+^ T cells in the stroma but not cancer nests through the production of CXCL12 since activated CD8^+^ T cells express high levels of CXCR4 [33]. The chemokine ligand/receptor has been demonstrated to be a strong chemoattractant for lymphocytes [34]. PSCs also induce T cell apoptosis and anergy by expressing galectin-1 [35]. PSCs may crosstalk with TAMs in PanIN, and these cell populations activate each other by secreting various soluble factors. This process may be the major mechanism of desmoplasia; interestingly, the deposition of collagen preferentially excludes TAMs [32].

### Infiltrating immune cells

The results of research on PDAC-infiltrating immune cells are often vague and controversial. Here, we summarize them concisely with a distinctive view.

#### Antitumour effector cells and immunodeficiency

Immune cells comprise nearly 50% of the PDAC cellular component [36], but only a few are antitumour effector cells. The low number of antitumour effector cells could possibly be attributed to the cells being disabled by several mechanisms (Fig. 2). Some studies have evaluated the function of tumour-associated neutrophils (TANs) in PDAC progression, which has been reviewed extensively [37]. In a recent clinical study, neutrophils were found to have an unexpected positive correlation with CD8^+^ T cells [38]; the correlation was surprising since these cells might play a role in excluding infiltrating T cells from PDAC tissue in mouse models [39, 40]. These controversial results may be interpreted as a function of the different neutrophil frequencies in humans and mice. The characteristics of natural killer (NK) cells within PDAC tumours have been investigated, but few reports describe the role of NK cells in normal and PDAC tissues [36, 41]. A study demonstrated that CD3^+^ T cells were the major immune cell type in PDAC, and the majority of resectable PDAC samples displayed intermediate to high levels of CD3^+^ T cell infiltration, which predominantly occurred in the stroma instead of the cancer cell nest centre [42]. CD3^+^ conventional T (Tconv) cells localize in tertiary lymphoid structures (TLSs) (Fig. 1) and co-localize with DCs, Treg cells, B cells, and high endothelial venules (HEVs). Localized proliferation, not merely migration, was shown to be a major source of activated T cells. Clonal T cell expansion was observed within the TLSs throughout the tumour lesions, indicating a tumour antigen-specific reaction within the TLSs [42]. In a subsequent study, heavy lymphocyte infiltration was observed in TLSs, but in situ proliferation was not observed [38]. Both of the above studies demonstrated a positive relationship between TLSs and OS in PDAC patients, suggesting that the potential antitumour response in PDAC is suppressed. Most of the tumour-infiltrating lymphocytes (TILs) displayed an antigen-experienced and memory-related phenotype [38, 42–44], which further supported this conclusion. The frequencies of CD4^+^ and CD8^+^ lymphocytes were variable among specimens; CD4^+^ T cells, especially CD4^+^ Tconv cells, were predominant, but CD8^+^ T cells were not [38, 42], suggesting a deficiency in the cytotoxic activity of CD8^+^ T cells. The accumulation of CD8^+^ T cells in PDAC is extremely variable; the frequency of CD8^+^ T cells among CD45^+^ leucocytes may be as high as 15–30% or less than 7%. These effector cells are functionally deficient, as they express various co-inhibitory molecules [38, 42].

**Fig. 3:**
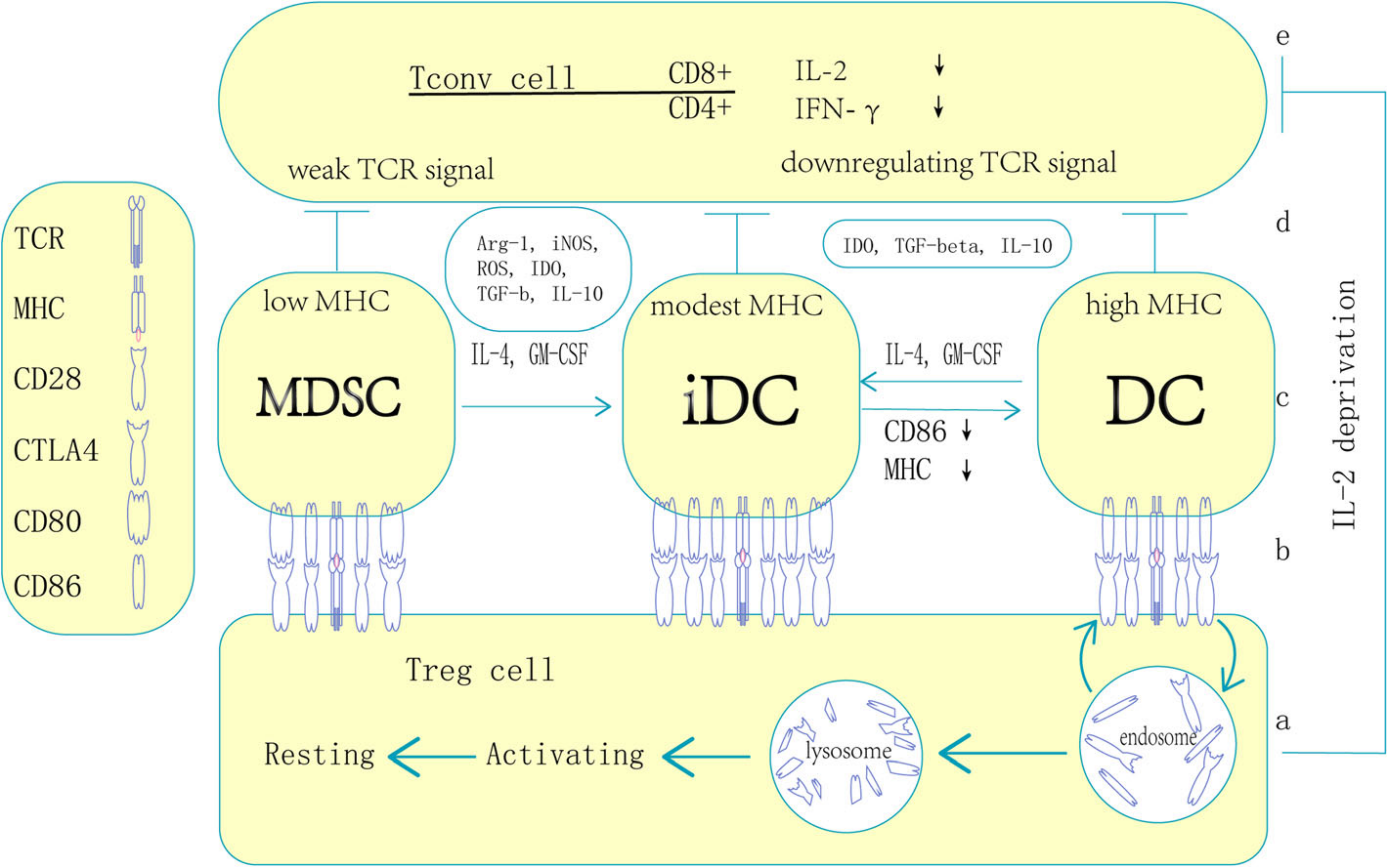
The mechanisms of Treg inhibit Tconv through APC. Treg and Tconv contact directly with the same APC and establish tumor specific suppressive TME. a: Treg capture and degradate CD86 on DC with CTLA4, the process occurs in LN/TLS and PanIN, activating Treg migrate to established tumor and transform to resting Treg and execute suppression; b: Treg (also Tconv) contact with APC through various pairs of ligand-receptor including of TCR/MHC, CD28/CD86, CD28/CD80, CTLA4/CD86, CTLA4/CD80, mature DC dominantly express high level of CD86 and combine with CD28 and CTLA4, MDSC preferentially express CD80 and combine with CTLA4, immature/inducible DC express both CD86 and CD80. Notably, MDSC express low level of MHC and enhance suppressive function of Treg with weak TCR signal, whereas DC express high level of MHC and promote Treg activation and proliferation; c: APC could transform each other with the effect of Treg and Tconv concordantly; d: APC inhibit Tconv through several soluble factors and induce Tconv anergy through weak/downregulating TCR signal; e: APC inhibit CD4+ Tconv directly and CD8+ Tconv indirectly mainly by downregulating IL-2 and IFN-γ et al., Treg cells could inhibit Tconv by depriving IL-2. Biophysical stability of CTLA4/CD28-CD80/CD86 polymer: CTLA4-CD80 > CTLA4-CD86 > CD28-CD86 > CD28- CD80

CD4^+^ and CD8^+^ T cells are subtly synchronized with each other within PDAC tumours; only patients with both CD4- and CD8-positive T cells have a significantly increased OS rate, and the CD4/CD8 double-positive T cell status is an independent prognostic factor [45, 46]. Among CD4^+^ Tconv cells, only the Th1 subset can facilitate the antitumour response, and the function of Th17 cells is controversial. Th2 cells are generally considered factors promoting tumour progression. Notably, Th2 cells are the major population of CD4^+^ T cells within PDAC tumours, and the Th2 CD4^+^ T cell number is higher than not only the Th1 CD4^+^ T cell number but also the FoxP3^+^ Treg cell number [47]. CD4^+^ T cells are inclined to polarize towards the Th2 phenotype, and this skewing is specific for carcinoembryonic Ag (CEA) [47]. These findings indicate that PDAC can induce TAA-specific immune impairment through CD4^+^ T cells. DCs in PDAC are generally functionally impaired. In a recent preclinical study, DCs were observed to abundantly infiltrate the tumour lesion, and DC accumulation increased as the disease progressed from PanIN to PDAC. However, the expression of the maturation marker MHC class II and the costimulatory molecules CD86 and CD40 was downregulated by Treg cells in a cell contact-dependent manner (Fig. 3) [48]. All of these molecules were indispensable for CD8^+^ T cell activation, and Treg cells could even suppress the in vivo expansion of tumour-infiltrating DCs [48]. PDAC epithelial cells can also exploit variable strategies to decrease the function of DCs, such as downregulating the expression of HLA-DR and CD40 to produce immature DCs and secreting DC-suppressing cytokines and chemokines [12, 49, 50]. Immature DCs can directly suppress the effector T cell response by expressing IDO [48]. DCs may execute antigen-specific suppressive functions by presenting tissue-specific antigens (TSAs) and even neoantigens to Treg cells to induce tumour-specific immunosuppression. Both DCs and Treg cells accumulate in TLSs with a high density of endothelial venules [38, 42], which are generally found in lymph nodes and responsible for antigen presentation. These facts highlight the possibility that tumour-specific immune tolerance exists in these structures through DC–Treg interactions.

#### Protumour regulatory cells and immunosuppression

Nearly all TAMs exhibit an M2 phenotype, identified by the surface markers CD163 and CD206 and cytokines, such as IL-10 and TGF-β, but they also display M1 characteristics [51]. TAM infiltration begins at a very early disease stage and persists in PDAC [36]. TAMs are generally located at the invasive front of the tumour (Fig. 1) [36, 52]. This process occurs in both murine and human PDAC and is accompanied by perineural invasion [53], lymphatic angiogenesis, lymph node metastasis [52, 54], cancer cell epithelial-mesenchymal transition (EMT) and extravasation [51]. Several factors can recruit monocytes to PDAC lesions and differentiate these cells into TAMs, including the hypoxic TME [55], vascular endothelial growth factor (VEGF)/epidermal growth factor receptor (EGFR) 2 axis [56], CCL2/CCR2 axis [14] and CSF1/CSF1R axis [57]. In an extensive study, Kaneda et al. [58] demonstrated that TAMs exploited numerous mechanisms to drive PDAC progression, including secreting immunosuppressive factors such as arginase-1 (Arg1) and TGF-β to inhibit antitumour CD8^+^ T cells and promoting PDAC desmoplasia and cancer cell metastasis via the chemotactic factor PDGF-BB. Therefore, the major role of TAMs in PDAC seems to be tightly regulating invasion and metastasis rather than inhibiting the immune response.

MDSCs are Gr1 and CD11b double-positive in mice and CD14-negative and CD11b-positive in humans. A subset of MDSCs express the granulocyte marker Ly6G at a high level and the monocyte marker Ly6C at an intermediate level; the other MDSC pool expresses high levels of Ly6C not Ly6G [59]. Therefore, MDSCs are categorized into two major subsets: granulocytic MDSCs (Gr-MDSCs) and monocytic MDSCs (Mo-MDSCs). MDSCs, especially Gr-MDSCs, are rare in the normal pancreas, and their accumulation increases progressively as the disease becomes invasive. MDSCs are widely dispersed throughout the tumour in invasive PDAC [36, 59]. PDAC cells highly express granulocyte macrophage colony-stimulating factor (GM-CSF), which was demonstrated to be a necessary and sufficient factor for functional and suppressive MDSC generation [39]. The function of MDSCs in PDAC was reviewed extensively in a previous publication [60]. Most investigators focus on the function of MDSCs in immunosuppression through the secretion of modulatory factors and direct contact with effector cells via checkpoint molecules. One important property of MDSCs worthy of emphasis is that although they are antigen presenting, they express low levels of the MHC II complex [59] and high levels of CD80 to induce antigen-specific immunosuppression via Treg cells (Fig. 3) [61]. Treg cells have T cell receptors (TCRs) with relatively high affinities for TSAs and constitutively express cytotoxic T lymphocyte-associated antigen 4 (CTLA-4), which preferentially binds with CD80 and outcompetes CD86 binding [62]. Gabrilovich et al. suggested that MDSCs might be involved in Treg cell differentiation [63]. These results indicate that TSA-specific and/or even neoantigen-specific immunosuppressive mechanisms mediated through the MDSC-Treg axis and antibodies against CD80 or CTLA-4 may have similar effects.

Treg cells have extensive interactions with various cells (Fig. 2), and the tight relationship between Treg and antigen-presenting-like cells has been repeatedly highlighted in numerous studies. However, overall conclusions are still obscure; Treg cells and antigen-presenting cells (APCs) cannot be defined restrictedly, and the molecular biophysical interactions between these two subsets of cells (particularly MHC/TCR, CTLA-4-CD28 and CD80-CD86 interactions) are controversial despite numerous researchers focusing on this field. We present an overview of the mechanism by which Treg cells inhibit Tconv cells via concordant contact with APCs (Fig. 3). Treg cells exert suppressive effects by recognizing self-TSAs presented by APCs but can inhibit effector cells in an antigen-independent fashion [64, 65]. Moreover, because the TCRs of Treg cells have higher affinities for epitopes than the corresponding TCRs of Tconv cells, Treg cells can recognize antigens at concentrations lower than those required for Tconv cell activation [65], suggesting that Treg cells may be activated by immature APCs with weak antigen presentation. Treg cells accumulate within tumours and tumour-draining lymph nodes at a very early stage in PanIN, and their numbers increase upon progression to PDAC [20, 48]. Upon the establishment of invasive tumours, Treg cells are generally localized within the TLSs with follicular DCs and HEVs (Fig. 1) [38, 42]. The prevalence of Treg cells is tightly correlated with the prognosis of PDAC [38, 48, 66–68] and generally has a negative relationship with patient OS. There are two major types of Treg cells: naturally occurring Treg cells (nTreg cells) derived from the thymus and resident in tissues and inducible Treg cells (iTreg cells) derived from naïve CD4^+^ T cells in the peripheral blood. PDAC cells produce CCL5 and VEGF to attract Treg cells through CCR5 [15, 16] and neuropilin-1 [48, 69]. Stromal cells recruit Treg cells by CXCL10 on PSCs [70] and CCL5 on MDSCs [71] through CXCR3 and CCR5, respectively (Fig. 2). These interactions may be the mechanism of iTreg cell accumulation since nTreg cells are generally resident cells. However, researchers have demonstrated that Treg cells accumulate in PDAC through proliferation and conversion in situ rather than via the infiltration of peripheral nTreg cells and naïve T cells [67]. Peripheral blood Treg cell depletion with an anti-CD25 antibody and functional inhibition do not affect the Treg cell frequency within tumours [72]. Localized proliferation is exploited by nTreg cells to drive accumulation within PDAC tissue at an early stage and is mediated by activation of tissue-resident nTreg cells by resident DCs through the presentation of self-antigens. Localized proliferation might also be the mechanism of iTreg cell accumulation within TLSs in which follicular DCs and HEVs are present. The function of Treg cells in PDAC immune editing also remains controversial, although most studies have demonstrated that Treg cells regulate CD4^+^ and CD8^+^ lymphocytes through monocyte-type cells. However, the pathway and target cells are not yet clear. In a pilot study, Qureshi et al. demonstrated that CTLA-4 molecules could capture and endocytose CD86 expressed on the cell surface, which resulted in CD86 degradation, and the subsequent activation of Treg cells prevented DCs from priming naïve T cells (Fig. 3) [73]. This may be the mechanism by which nTreg cells inhibit tissue-resident DCs in early PanIN lesions since tissue-resident DCs rarely express CD86 rather than CD80. On the other hand, Treg cells may regulate infiltrating CD4^+^ cells rather than CD8^+^ T cells in PDAC through the CTLA-4/CD80 pathway by contacting MDSCs because blockade of CTLA-4 on Treg cells or blockade of CD80 on MDSCs was shown to produce the same results [72]. Based on these observations, MDSCs appear to have a high probability of being monocyte-type cells targeted by iTreg cells in invasive PDAC. Treg cells express TCRs that recognize self-TSAs and may be activated by self-TSAs in the presence of APCs [74, 75]. This property of Treg cells may be exploited by cancer cells and immature APCs to produce immune tolerance. It has been previously demonstrated that immature APCs can preferentially induce Treg cells [76, 77]. Immature APCs may have a better potential to facilitate the suppressive function of Treg cells than mature APCs because of their higher expression levels of CD80 [62], which generally forms a dimer and preferentially binds with CTLA-4 molecules, which are constitutively expressed on Treg cells (Fig. 3). Targeting tissue-specific Treg cells and/or blocking the interaction between Treg cells and monocyte-like cells may be an interesting direction of research for PDAC immunotherapy.

## mAb therapy for PDAC

mAb-based therapy has been used as an established treatment strategy for multiple solid tumours for decades. The functional mechanisms of mAbs in cancer therapy are limited to not only the direct killing of cells through antibody-dependent cellular cytotoxicity (ADCC) and similar pathways but also to the regulation of the immune microenvironment by blocking the corresponding signalling pathway, reversing immunosuppression and enhancing the activity of antitumour effector cells. mAbs could even be used for the delivery of various therapeutic reagents (Table 1).

**Table 1:** mAb-Based Therapies Targeting Non-Immune Cells for PDAC

mAb	Condition	Target	Cells	Mechanism	Status	Reference
SS1(dsFv)-PE38 (SS1P)	An anti-mesothelin Fv genetically fused with a truncated pseudomonas exotoxin, PE38	Mesothelin	Cancer cells	PE38 is internalized into and kills cancer cells through inhibition of protein synthesis by ADP ribosylation and inactivation of elongation factor 2	Clinical trials	NCT01362790; NCT00006981
MORAb-009	Heavy and light chain variable regions of a mouse anti-mesothelin single chain Fv grafted to human IgG1 and κ constant regions	Mesothelin	Cancer cells	Inhibits the adhesion between cells expressing mesothelin and MUC16 as well as mediates ADCC	Clinical trials	NCT00570713; NCT00325494; NCT01521325; NCT01413451
BAY94–9343	An ADC consisting of a human anti-mesothelin antibody conjugated to a tubulin inhibitor, DM4	Mesothelin	Cancer cells	Binds to human mesothelin and induces antigen internalization	Clinical trials	NCT03023722; NCT03816358; NCT03102320
GP1.4	Monoclonal antibody	MUC1	Cancer cells	Induces MUC1 internalization and inhibits ERK signalling, resulting in the suppression of PDAC cell proliferation and migration	Preclinical study	[78]
A novel produced monoclonal antibody	Monoclonal antibody	MUC1-C	Cancer cells	Induces MUC1 internalization and inhibits ERK signalling, resulting in the suppression of PDAC cell proliferation and migration	Preclinical study	[79]
A.4.6.1	Murine-derived monoclonal antibody	VEGF	Cancer cells	Inhibits the angiogenesis of tumours	Preclinical study	[80]
Erlotinib	Monoclonal antibody	EGFR	Cancer cells	Inhibits the angiogenesis of tumours	Clinical trials	NCT00810719; NCT02154737; NCT02694536; NCT01782690; NCT00614653; NCT00640978; NCT00565487; NCT00313560; NCT01608841; NCT01303029
Bevacizumab	Monoclonal antibody	VEGF	Cancer cells	Inhibits the angiogenesis of tumours	Clinical trials	NCT00614653; NCT00047710; NCT00460174; NCT00365144; NCT00410774; NCT00417976; NCT00112528; NCT00602602; NCT00126633; NCT00366457
9E1	Monoclonal antibody	AnxA6	Cancer cells, potential stromal cells	Reduces the expression of MMP-9 and/or interferes with ERK and MEK signalling	Preclinical study	[81]
Demcizumab	Monoclonal antibody	DLL4	CSCs	Decreases CSC frequency and interferes with angiogenesis	Clinical trials	[82]; NCT01189929; NCT02289898
Clivatuzumab (PAM4)	^131^I-labelled,^90^Y-labelled	MUC1	Cancer cells	Radioimmunotherapy	Clinical trials	[83]
TF10 (A humanized recombinant structure)	^90^Y-labelled	MUC1	Cancer cells	Radioimmunotherapy	Preclinical study	[83]
C595	^213^Bi-labelled	MUC1	Cancer cells	Radioimmunotherapy	Preclinical study	[83]
CC49	^131^I-labelled	TAG-72	Cancer cells	Radioimmunotherapy	Preclinical study	[83]
EGFR antibody	^177^Lu-labelled	EGFR	Cancer cells	Radioimmunotherapy	Preclinical study	[83]
Trastuzumab	^213^Bi-labelled	HER2	Cancer cells	Radioimmunotherapy	Preclinical study	[83]
TNT3 antibodies	^213^Bi-labelled	Single-strand DNA and RNA	Released from necrotic cells	Radioimmunotherapy	Preclinical study	[83]
KAb201	^131^I-labelled	CEA	Cancer cells	Radioimmunotherapy	Clinical trial	[83]
MN-14	^131^I-labelled	CEA	Cancer cells	Radioimmunotherapy	Clinical trial	[83]
TCMC-Trastuzumab	^212^Pb-labelled	HER2	Cancer cells	Radioimmunotherapy	Clinical trial	[83]
059–053	^90^Y-labelled	CD147	Cancer cells	Radioimmunotherapy	Preclinical trial	[84]
376.96	^212^Pb-labelled	B7-H3	Cancer cells	Radioimmunotherapy	Preclinical trial	[85]
TIBs derived IgG	Unidentified multiclonal antibody	G12 mutation-derived epitopes	Cancer cells	Targets mutation-derived and personalized antigens	Preclinical study	[86]

In this chapter, we focus on mAb therapy directed against cancer and stromal cells. Mesothelin (MSLN) is extensively expressed in several solid tumours and in almost 100% of PDAC cells [87]. MSLN plays a critical role in the development of pancreatic cancer, especially at an early stage, and in peritoneal metastasis by binding with its single ligand MUC16; however, the intracellular mechanism remains unclear [88]. Furthermore, overexpression of MSLN is associated with poor outcomes for PDAC patients [89]. Several preclinical and clinical trials of MSLN-targeted mAb-based therapy have been summarized by several reviews [90–92]. In brief, the functional mechanisms of anti-MSLN mAb include not only ADCC but also alteration of intracellular signalling in cancer cells through endocytosis. This phenomenon has been exploited to deliver cytotoxins to kill cancer cells [93]. Anti-MSLN antibodies can also block the binding of MSLN with MUC16 and inhibit the expansion and metastasis of cancer cells [88]. MORAb-009 is a humanized antibody known as amatuximab. Baldo demonstrated that amatuximab exerts therapeutic efficacy by inducing ADCC and inhibiting the binding of MSLN with MUC16 [94]. Hassan, Fujisaka and their colleagues successively reported two phase I clinical studies including PDAC and other solid tumours expressing MSLN. They demonstrated the safety of amatuximab but observed no apparent objective responses despite stable disease occurring in some patients [95, 96].

MUC1 is restricted to apical surface expression on normal epithelial cells [97] and is overexpressed in approximately 90% of PDAC cells [98] on the basolateral membrane [97]. Biochem and colleagues demonstrated that an antibody similar to the anti-MUC1 antibody GP1.4 could trigger the internalization of EGFR on PDAC cells. This process could inhibit ERK signalling and result in the inhibition of cancer cell proliferation and migration [78], but the mechanism was unclear. Wu et al. [79] recently reported that MUC1-C, an isoform of MUC1, was highly expressed in 60.6% of human PDAC tissue samples compared to normal tissue samples. They used the same anti-MUC1 antibody on human pancreatic cell lines and a xenograft mouse model and demonstrated that the anti-hMUC1 antibody could pass through the membrane, inactivate MUC1 signalling and then suppress tumour growth in vivo. Since GP1.4 can be internalized by cancer cells, whether it can be exploited as a carrier of a cytotoxin would be an interesting investigation.

VEGF can promote vascularization in cancer lesions, and although PDAC does not have high vessel density, the cancer cells aberrantly express VEGF. This conclusion is supported by an early preclinical study that used the murine-derived anti-VEGF antibody A.4.6.1 to supress tumour growth [80]. Another anti-VEGF antibody, bevacizumab, has been the subject of multicentre-based investigations in combination with chemotherapy, but the results have not yet been published. Treatment combining the anti-EGFR antibody erlotinib with Gem was recently carefully assessed, and mild efficacy and tolerable adverse effects were concluded (Table 1) [99, 100].

AnxA6 is expressed in almost all PDACs by CAFs and localizes at the invasive front of the tumours, where it forms a complex structure with LDL receptor-related protein 1 and thrombospondin and participates in crosstalk between cancer cells and the stroma. The structure has shown strong correlations with cancer cell survival and perineural invasion [101]. O’Sullivan et al. isolated a novel antibody against AnxA6, 9E1, and demonstrated in an ex vivo experiment that the antibody could reduce the invasive capacity of pancreatic cancer cells by reducing MMP-9 expression and modulating ERK and MEK signalling [81].

Delta-like ligand 4 (DLL4) may be another possible mAb target for PDAC treatment since the DLL4 signalling pathway is important for PDAC cancer stem cell (CSC) survival. Demcizumab is a humanized anti-DLL4 antibody that has the potential to reverse chemotherapy resistance, and a study showed that demcizumab combined with paclitaxel and Gem was safe but not efficacious [82]. Two clinical trials on the use of demcizumab for PDAC treatment were completed recently, but the results have not yet been published (Table 1).

Antibodies or antibody fragments can also be conjugated with radioisotopes to deliver localized radiotherapy, known as radioimmunotherapy, and is emerging as an important selection for PDAC patients [83]. Recently, CD147 [84] and B7-H3 [85] were explored as targets of radioimmunotherapy for cancer cells and CSCs, respectively, with a ^90^Y-labelled antibody (059–053) and a ^212^Pb-labelled antibody (376.96) and investigated in preclinical experiments; both achieved promising results and demonstrated potential therapeutic efficacy for PDAC (Table 1).

Mutation of the Kras gene may be a promising target for mAbs in PDAC since more than 90% of PDAC cases bear a mutation at position G12 [102]. In a pilot study, Meng et al. demonstrated that tumour-infiltrating B cell (TIB)-derived IgGs could recognize most G12 mutations occurring in PDAC and noted that TIBs might be a source of antitumour antibodies targeting neoantigens [86]. This study established a novel way to produce neoantigen-targeting antibodies for personalized mAb immunotherapy.

## Strategies reversing immunosuppressive mechanisms

### ICI therapy

Only approximately 4% of all PDAC cells, including cancer cells (5.5% ± 1.1), CD163^+^ TAMs (9.3% ± 3.6) and CAFs, express PD-L1 [38]. Although the majority of PDAC cases show intermediate to high numbers of infiltrating T cells, CD4^+^ T cells, rather than CD8^+^ T cells, are the main component [38, 42]. The objective response of malignancy to ICI therapy is positively associated with the mutational burden, which is relatively low in PDAC [103, 104]. All of these factors indicate a dismal response to ICI therapy by PDAC compared to other solid tumours [104–107]. Investigators are trying to improve the effect of ICI therapy through different approaches. GM-CSF-secreting tumour cells (GVAX) can significantly upregulate PD-L1 expression and improve the effect of anti-CTLA-4 and anti-PD-1/PD-L1 antibodies [17, 108]. Oncolytic virotherapy [109], chemotherapy and radiotherapy [110, 111], a CSF1 blockade [57], an anti-IL-6 antibody [112], a CXCL12/CXCR4 axis inhibitor and stromal cell depletion [113] have also been tested to enhance the efficacy of ICI therapy on PDAC. Among these efforts, the combination of ICI therapy and chimeric antigen receptor (CAR) T cell infusion may hold the most promise [114, 115], as this strategy can simultaneously increase the number of tumour-targeting effector cells and prevent infused cell anergy.

### Strategies targeting immunosuppressive cells

#### Treg cells

Chemotherapy reverses immunological tolerance for a prolonged period [116], and the mechanism was demonstrated by selectively depleting Treg cells [117]. Cyclophosphamide (Cy) is the most commonly used agent to deplete Treg cells to enhance cytotoxic and helper T cell responses [118]. Treg cells lack the ATP-binding cassette (ABC) transporter, which can extrude Cy out of cells, causing Treg cells to be more susceptible to Cy than other T cells [119]. Gem is another chemotherapeutic drug selectively capable of depleting Treg cells. Shevchenko et al. observed that in a mouse model, the depletion of local Treg cells with a low dose of Gem significantly improved the modest survival rate without affecting tumour growth or metastasis [67]. While Beatty et al. demonstrated that the depletion of Treg cells in the peripheral blood did not affect the Treg cell frequency in the tumour lesion and had no effect on tumour progression, a CD40 agonist used in combination with Gem decreased the Treg cell numbers and the accumulation of CD4^+^ and/or CD8^+^ cells in xenograft and/or orthotopic tumours [110], indicating that Gem, which can deplete tumour-infiltrating Treg cells, may restore the antitumour effects of CD40 agonists and ICIs. These results suggested that tumour-infiltrating Treg cells rather than circulating Treg cells accounted for the overall Treg function; targeting local proliferating/accumulating Treg cells but not peripheral Treg cells might be more advantageous and have fewer adverse effects on the immune system. Treg cell depletion can also enhance the effect of a PDAC vaccine. Lei Zheng and colleagues treated PDAC patients with a low dose of Cy in combination with GVAX and observed Cy-dependent Treg cell depletion and lymphoid aggregate formation in the PDAC TME. In addition, decreased Treg cell numbers in the lymphoid aggregates not only enhanced existing effector T cell activation but also facilitated more effector T cell trafficking into PDAC tumours [120]. Even premalignant PanIN lesions could benefit from Treg cell depletion; Treg cell depletion combined with the LM-Kras vaccine (attenuated *Listeria monocytogenes* strain expressing KrasG12D) could recruit CD4^+^ and CD8^+^ effector T cells to the premalignant lesion and inhibit PanIN progression. This strategy could also enhance the recruitment of Gr-1^+^ cells but repolarize them into an antitumour phenotype to enable cytokine production and the induction of an inflammatory response [121]. This study further verified the tight correlation between Treg cells and MDSCs.

#### MDSCs and TAMs

The subtle distinction between Gr-MDSCs and Mo-MDSCs should be noted. In a preclinical study to test the potential of targeting MDSCs, Stromnes et al. demonstrated an extensive effect of depleting Gr-MDSCs on the prognosis of PDAC patients and determined the rational mechanism. They selectively depleted Gr-MDSCs with the anti-Ly6G mAb 1A8. Compared with untreated mice, treated mice showed a 4- to 5-fold increase in Mo-MDSC numbers in the spleen and PDAC lesions, and the gross number of tumour-infiltrating CD45^+^ cells increased approximately 2-fold in 1A8-treated mice [59]. Further study indicated that the numbers of proliferating and activated CD8^+^ T cells with high granzyme B levels increased absolutely, and these cells were found in not only the stroma but also in the proximity of tumour cells. Decreased stromal matrix deposition and integrity, increased caspase-3-positive tumour cell numbers and blood vessels were observed in 1A8-treated tumours [59]. There was no observed reduction in tumour size due to an influx of tumour-reactive effector cells, a phenomenon known as tumour pseudoprogression [122]. The compensatory increase in Mo-MDSCs synchronized with the depletion of Gr-MDSCs was remarkable, and a similar result was reported in another study in which the decrease in TAMs/Mo-MDSCs was accompanied by an increase in Gr-MDSCs. The checks and balances between Gr-MDSCs and Mo-MDSCs may indicate some therapeutic value; although these cells share some similar phenotypic molecules and show similar suppressive functions, these two myeloid cell subsets might have very distinct final fates and should be handled separately. TAMs are a pool of cells with heterogeneous functions and phenotypes, and their versatile plasticity allows their transformation into each other according to the local conditions. Both the CSF1/CSF1R and CCL2/CCR2 axes are critical for the accumulation and differentiation of TAMs from their progenitors in the blood. A CSF1/CSF1R blockade can not only decrease the number of TAMs in PDAC lesions but also reprogram TAMs to enhance their antigen-presenting ability, resulting in enhanced antitumour T cell responses [57]. In a contemporary preclinical study [123], Mitchem et al. investigated an axis-targeting treatment combined with chemotherapy and demonstrated that CCR2 and/or CSF1R inhibitors displayed only modest effects. Gem alone could increase the number of TAMs in PDAC lesions, and CCR2 and/or CSF1R inhibitors could reverse this increase and dramatically reduce tumour masses. In addition, the researchers observed significant CD4^+^ and CD8^+^ T cell infiltration and decreased Treg cell infiltration after treatment. Remarkably, they found that a CCR2 and/or CSF1R blockade could decrease the numbers of both TAM and Mo-MDSC, which was potentially the result of a phenotypic overlap between these two monocyte subsets. However, a modest increase in Gr-MDSC numbers was observed, which was potentially due to a compensatory relationship between the two types of MDSCs. Specifically, blocking either CCR2 or CSF1R could disrupt this interaction and reverse chemotherapy resistance [123]. TAMs generally localize at the invasive front of PDAC lesions and are involved in angiogenesis and EMT, which are important for cancer cell invasion and metastasis. Investigations of methods to reverse or inhibit this function of TAMs would be interesting.

## Strategies enhancing the antitumour response

### Costimulatory molecule agonists

In a pilot study, Beatty et al. demonstrated an unexpected function of a CD40 agonist, as treated F4/80^+^ macrophages in the peripheral blood were activated and infiltrated tumour lesions. However, although the expected T lymphocyte infiltration was not observed, the PDAC stroma was destroyed, and cancer cells were killed by the infiltrating macrophages [124]. The researchers further demonstrated that this agonist of CD40 upregulated the expression of MHC class II and CD86, suggesting an enhanced antigen-presenting ability of the macrophages. Nevertheless, T cells did not infiltrate tumours and remained in the peripancreatic lymph nodes adjacent to the tumours, suggesting that an additional mechanism excluded these antitumour effector cells. In a subsequent study [125], the same team found that the agonist of CD40 induced heavy T cell infiltration into tumours upon combination with Gem and resulted in CD4^+^ and/or CD8^+^ T cell-dependent tumour regression. They explained the controversial results by concluding that circulating macrophages may have dual roles in regulating immunoreactivity in PDAC but did not interpret the role of Gem in the treatment. Gem combined with the CD40 agonist could induce tumour regression even after circulating macrophages were depleted [125]. This result suggested that the chemotherapeutic agent in the experiment targeted some unknown immunosuppressive cells that could exclude effector T cells. Rationally, these cells were probably Treg cells since Gem has been demonstrated to be a potent Treg cell-depleting agent in PDAC [67]. In a multicentre phase I clinical study by Beatty and his collaborators, an agonistic anti-CD40 antibody was applied in combination with Gem for PDAC treatment; while only a mild effect was observed, the safety of the combination was established [126]. In addition, the CD40 agonist and Gem combination could also reverse resistance to ICI therapy via promoting the accumulation of robust antitumour CD8^+^ T cells in PDAC tumours [110]. These results potentially demonstrate that the combination of reprogramming macrophages to enhance their antigen-presenting ability with Treg cell depletion and ICI administration is a promising approach. The stromal destruction observed with both Gr-MDSC depletion (increase in tumour-infiltrating Mo-MDSC numbers) [59] and TAM reprogramming [124] indicates that Mo-MDSCs and TAMs share an overlapping role.

### ACT

ACT is a very active field of investigation in PDAC immunotherapy and is performed using lymphocytes with or without gene editing and TILs (Table 2). Substantial progress has been made over the last three years regarding PDAC.

**Table 2:** ACT Clinical Trials for PDAC

NCT number	Status	Intervention	Phase	Duration	Location
NCT03008304	Recruiting	High-activity natural killer	Phase 1 Phase 2	December 2016–December 2019	Fuda Cancer Institute of Fuda Cancer Hospital, Guangzhou, Guangdong, China
NCT03267173	Recruiting	Chimeric antigen receptor T cell	Phase 1	June 15, 2017-June 2019	Harbin Medical University, Harbin, Heilongjiang, China
NCT03180437	Recruiting	γδ T cell	Phase 1 Phase 2	June 15, 2017-June 15, 2020	Biotherapy Centre at Fuda Cancer Hospital, Guangzhou, Guangdong, China
NCT03136406	Active, not recruiting	aNK	Phase 1 Phase 2	August 14, 2017-December 2018	Chan Soon-Shiong Institute for Medicine, El Segundo, California, United States
NCT03329248	Active, not recruiting	haNK	Phase 1 Phase 2	November 6, 2017-December 2019	Chan Soon-Shiong Institute for Medicine, El Segundo, California, United States
NCT02929797	Recruiting	CD8 + NKG2D+ AKT cells	Early Phase 1	August 2016–August 2019	Shanghai General Hospital, Shanghai, China
NCT03323944	Recruiting	huCART-meso cells	Phase 1	September 15, 2017-September 2021	University of Pennsylvania, Philadelphia, Pennsylvania, United States
NCT02718859	Unknown	NK cells	Phase 1 Phase 2	March 2016–March 2017	Central Laboratory at Fuda Cancer Hospital, Guangzhou, Guangdong, China
NCT01781520	Completed	DC-CIK	Phase 1 Phase 2	June 1, 2013-June 13, 2017	Capital Medical University Cancer Center, Beijing, Beijing, China
NCT03387098	Active, not recruiting	haNK	Phase 1 Phase 2	January 2, 2018-December 2019	Chan Soon-Shiong Institute for Medicine, El Segundo, California, United States
NCT00003780	Unknown	Tumour-infiltrating lymphocytes	Phase 2	December 1998, the last update posted is December 19,2013	Meyer Pharmaceuticals, LLC, Irvine, California, United States
NCT02529579	Recruiting	iAPA-DC/CTL	Phase 1 Phase 2	June 2015–December 2019	Changhai Hospital, Second Military Medical University, Shanghai, China
NCT03638193	Recruiting	CAR-T-meso cells	Not Applicable	July 11, 2018-February 1, 2022	Nanjing First Hospital, Nanjing, Jiangsu, China
NCT03013712	Recruiting	CAR-T cells targeting EpCAM	Phase 1 Phase 2	January 2017–December 2020	IEC of Chengdu Medical College Chendu, China
NCT01583686	Terminated	Anti-mesothelin CAR transduced PBL	Phase 1 Phase 2	May 4, 2012-December 17, 2018	National Institutes of Health Clinical Center, 9000 Rockville Pike, Bethesda, Maryland, United States
NCT01420874	Active, not recruiting	EGFR2Bi-coated T cells	Phase 1	August 2011–June 2019	Barbara Ann Karmanos Cancer Institute, Detroit, Michigan, United States
NCT03638206	Recruiting	Mesothelin targeting CAR-T cells	Phase 1 Phase 2	March 1, 2018-March 1, 2023	The First Affiliated Hospital of Zhengzhou University, Zhengzhou, Henan, China
NCT00909558	Suspended	Autologous natural killer/natural killer T cell	Phase 1	May 2009, the last update posted is February 24, 2010	Envita Medical Centers, Scottsdale, Arizona, United States
NCT02839954	Unknown	Anti-MUC1 CAR-pNK cells	Phase 1 Phase 2	July 2016–July 2018	PersonGen BioTherapeutics (Suzhou) Co., Ltd., Suzhou, Jiangsu, China
NCT00019084	Completed	Tumour-infiltrating lymphocytes	Phase 2	February 1996–May 2003	Medicine Branch, Bethesda, Maryland, United States
NCT03093688	Recruiting	iNKT cells and CD8+ T cells	Phase 1 Phase 2	March 1, 2017-December 31, 2019	Shanghai Public Health Clinical Center, Shanghai, Shanghai, China
NCT01174121	Recruiting	Young tumour-infiltrating lymphocytes	Phase 2	August 26, 2018-December 27, 2024	National Institutes of Health Clinical Center, 9000 Rockville Pike, Bethesda, Maryland, United States
NCT01801852	Unknown	NKT cells	Not Applicable	January 2013–June 2017	Biotherapeutic Department of Chinese PLA General Hospital, Beijing, Beijing, China
NCT02465983	Completed	CAR-T-meso-19 T cells	Phase 1	May 2015–November 2017	University of California, San Francisco, San Francisco, California, United States
NCT02757391	Not yet recruiting	CD8+ T cells	Phase 1	December 31, 2018-December 31, 2022	M D Anderson Cancer Center, Houston, Texas, United States
NCT03269526	Active, not recruiting	Anti-CD3 x anti-EGFR bispecific antibody (EGFRBi) armed activated T cells (EGFR BATs)	Phase 1 Phase 2	July 28, 2017-June 1, 2022	University of Virginia, Charlottesville, Virginia, United States
NCT03682744	Recruiting	Anti-CEA CAR-T cells	Phase 1	September 13, 2018-September 2019	Rutgers Cancer Institute of New Jersey, New Brunswick, New Jersey, United States Roger Williams Medical Center, Providence, Rhode Island, United States
NCT03407040	Enrolling by invitation	Generation of cancer antigen-specific T cells from human-induced pluripotent stem cells (iPSCs)	Phase:	January 30, 2018-December 31, 2030	National Institutes of Health Clinical Center, Bethesda, Maryland, United States
NCT03745326	Recruiting	Anti-KRAS G12D mTCR PBL	Phase 1 Phase 2	January 16, 2019-December 1, 2028	National Institutes of Health Clinical Center, Bethesda, Maryland, United States
NCT03190941	Recruiting	Anti-KRAS G12V mTCR	Phase 1 Phase 2	September 21, 2017-June 29, 2028	National Institutes of Health Clinical Center, Bethesda, Maryland, United States
NCT02850536	Active, not recruiting	Anti-CEA CAR-T cells	Phase 1	February 1, 2017-August 2018	University of Colorado Hospital, Aurora, Colorado, United States Roger Williams Medical Center, Providence, Rhode Island, United States

#### ACT with genetically engineered cells

CAR-engineered T cell (CAR-T) ACT for PDAC was very recently thoroughly reviewed [127–131]. Various artificial gene-design strategies targeting the cancer stroma and overcoming immunosuppressive factors have been explored to improve the effect of CAR-T ACT on PDAC. Rataj et al. genetically engineered ovalbumin (OVA)-specific CD4^+^ and CD8^+^ T cells with a PD-1-CD28 fusion protein. They observed significant synergy between the two cell populations correlating with the number of CD4^+^ T cells, indicating that the PD-1/PD-L1 suppressive signal was reversed and that the helper function of CD4^+^ T cells and antitumour effect of CD8^+^ T cells were enhanced [132]. Mohammed et al. performed a similar experiment [133] wherein they engineered the T cell population with two genes simultaneously, a first-generation PSCA-specific CAR and an inverted cytokine receptor (ICR) with an IL-4 extracellular domain and an IL-7 intracellular domain to yield CAR/ICR T cells. CAR/ICR T cells could reverse the IL-4-derived inhibitory signal to the T cell proliferation signal and showed enhanced antitumour activity. Genetically engineered TCR T cell (TCR-T) infusion is another ACT strategy. Stromnes et al. conducted ground-breaking research in this field, wherein a series of pilot and extensive experiments generated valuable data [134]. They screened a TCR for an endogenous unmutated MSLN epitope, which worked in an MHC class I-independent manner. TCR-Ts accumulated preferentially in orthotopic PDAC lesions and induced cancer cell death as well as stromal remodelling. Serial TCR-T infusion was performed, and improved survival was observed without increased toxicity [134].

#### TILs and neoantigens

CD3^+^ T cells were shown to comprise up to 90% of all tumour-infiltrating cells [41] and for almost all CD45RO^+^ memory cells [38, 42–44]. Recently, Hall and Meng reported the successful extraction of TILs from PDAC specimens and the expansion of these cells in vitro [135, 136]. However, they used different protocols to isolate and expand the TILs from tumour fragments. Hall et al. used medium containing a high dose of IL-2 and obtained TILs composed primarily of CD4^+^ T cells, while Meng et al. cultured fragments with medium containing the cytokines IL-2, IL-15, and IL-21 and expanded TILs composed primarily of CD8^+^ T cells. Both research teams demonstrated autologous tumour cell killing activity in an HLA-dependent manner. In a pilot study [42], Poschke et al. observed clonal tumour-reactive T cell expansion in PDAC, and they isolated and expanded TILs with a success rate similar to that achieved in melanoma. The authors reported that ex vivo culture appeared to reverse the exhausted phenotype of the freshly isolated TILs, but the proportion of tumour-reactive T cells was very low in the final pools, and these cells displayed no effect against an autologous PDAC xenograft. The researchers further interpreted the phenomenon of TCR repertoire alteration during ex vivo expansion. The regulatory cells within TIL populations should be carefully considered because they may exist in the fragment culture for a long time and bias the nonspecific expansion of TILs. Since TCR repertoire alteration might be the major hurdle for TIL treatment in PDAC, the identification of tumour-specific TCRs and/or TIL clones may be an alternative approach. In a very recent study, Meng et al. reported the production of three TIL cell lines and two autologous tumour cell lines; they screened, sequenced and synthesized mutation-derived neopeptides and observed neoantigen-specific tumour killing in an HLA-dependent fashion. They demonstrated the presence of neoantigen-specific TIL clones in both CD8^+^ and CD4^+^ T cell pools, which functioned in HLA class I- and HLA class II-dependent manners, respectively. Importantly, they reported that peripheral blood mononuclear cells (PBMCs) as well as TILs could be used to screen neoantigens. These results pave the way for highly specific and personalized ACT [137] since targeting personalized mutations has been demonstrated to be a durable approach for the treatment of metastatic solid tumours with a relatively low mutation burden [138].

### Vaccines

The vaccines used for PDAC therapy are diverse and employ very different mechanisms (Table 3). Briefly, there are three major vaccine platforms for PDAC: DC-based vaccines, tumour cell-based vaccines and bacterium-based vaccines. DCs are the most common platform, and DC-based vaccines have been tested in numerous clinical trials and thoroughly reviewed [139, 140]. Another PDAC vaccine platform is the whole-tumour cell vaccine platform using autologous and/or allogeneic cancer cells with or without genetic editing. GVAX is a whole-cell vaccine system used extensively for treating various cancers, including PDAC. GVAX vaccines for PDAC are derived from two pancreatic cancer cell lines engineered with the GM-CSF gene; these vaccines can be injected intradermally and secrete high levels of GM-CSF to attract APCs and promote their maturation. The vaccines have been demonstrated to be safe but to have modest effects [141, 142]. It should be noted that GM-CSF alone is not sufficient for APC maturation, and the simultaneous presence of IL-4 is indispensable. Algenpantucel-L is another whole-cell vaccine consisting of two pancreatic cancer cell lines genetically engineered to express α-galactosyl (α-gal) epitopes on membrane glycoproteins and glycolipids [143]; these epitopes are not expressed in human cells [144] and induce complement- and antibody-dependent cytotoxicity since there are large amounts of anti-α-gal antibodies in human serum [145]. Algenpantucel-L combined with chemotherapy moderately improved the 1-year OS rate of patients with resectable PDAC without severe adverse effects [143]. Tanemura and Doki et al. subsequently produced whole-cell vaccines expressing α-gal epitopes based on cancer cell lines and tumour lysates separately and demonstrated therapeutic potency in preclinical studies; notably, both vaccines could target both cancer cells and CSCs [146, 147]. Recently, a bacterium-based vaccine, CRS-207, was developed that comprises a recombinant live-attenuated *Listeria monocytogenes* strain engineered to secrete MSLN into the cytoplasm of infected APCs. This strategy could not only enhance the ability of APCs but also target an antigen universally expressed by PDAC. It has been demonstrated to be safe, and the combination of GVAX and CRS-207 has shown a survival benefit [148, 149]. The fact that the epitopes used to enhance effector cell antitumour reactivity can also be presented to Treg cells and result in tumour-specific immune tolerance is an important phenomenon that should be emphasized and can be used to interpret the mild effect of whole-cell and DC vaccines [150, 151]. How to overcome suppressive cells, especially tumour antigen-specific Treg cells, is a critical issue that needs to be resolved [152].

**Table 3:** Vaccine Clinical Trials and Mechanisms in PDAC

NCT Number	Status	Immunogen	Condition	Mechanism	Phase
NCT02405585	Terminated	Algenpantucel-L vaccine	Mfolfirinox, radiation and gemcitabine	Human pancreatic cancer cells engineered with a mouse Algenpantucel-L gene to make the cancer cells foreign	Phase 2
NCT01072981	Completed	Algenpantucel-L vaccine	Gemcitabine or 5FU chemoradiation	Human pancreatic cancer cells engineered with a mouse Algenpantucel-L gene to make the cancer cells foreign	Phase 3
NCT01836432	Unknown	Algenpantucel-L vaccine	FOLFIRINOX, 5-FU chemoradiation, gemcitabine, capecitabine, NabPaclitaxel	Human pancreatic cancer cells engineered with a mouse Algenpantucel-L gene to make the cancer cells foreign	Phase 3
NCT03114631	Enrolling by invitation	Dendritic cells pulsed with tumour lysate, dendritic cells pulsed with MUC-1/WT-1 peptides	NO	Increase MUC1/WT1-specific T cell response	Phase 1 Phase 2
NCT00547144	Completed	Intratumoural autologous dendritic cell vaccination	Gemcitabine and stereotactic radiosurgery	Increase tumour-specific T cells	Phase 2
NCT00795977	Unknown	Intratumoural autologous dendritic cell vaccination in combination with OK-432	NO	Induce tumour antigen-specific T cell response and tumour festering reaction	Phase 1 Phase 2
NCT03136406	Active, not recruiting	GI-4000	Cyclophosphamide, oxaliplatin, capecitabine, fluorouracil, leucovorin, nab-paclitaxel, bevacizumab, avelumab, ALT-803, aNK, and ETBX-011	Vaccine derived from recombinant *Saccharomyces cerevisiae* yeast expressing mutant Ras proteins to induce the Ras protein-specific reaction	Phase 1 Phase 2
NCT03329248	Active, not recruiting	GI-4000	ETBX-011, haNK, avelumab, bevacizumab, capecitabine, cyclophosphamide, fluorouracil, leucovorin and 4 more	Induce mutant Ras protein-specific antitumour reaction	Phase 1 Phase 2
NCT03387098	Active, not recruiting	GI-4000	Aldoxorubicin HCl, ALT-803, ETBX-011, haNK for infusion, avelumab, bevacizumab, capecitabine, cyclophosphamide, fluorouracil and 5 more	Induce mutant Ras protein-specific antitumour reaction	Phase 1 Phase 2
NCT00837135	Withdrawn	GI-4000	Activated T cells	Induce mutant Ras protein-specific antitumour reaction	Phase 1
NCT00002773	Completed	Allogeneic tumour cell vaccine	Recombinant IFNγ, sargramostim and cyclophosphamide	Induce tumour-specific reaction between patients sharing some TSAs	Phase 2
NCT00003025	Completed	HSPPC-96	NO	Autologous tumour-derived pg96 heat shock protein complex to induce an antitumour reaction	Phase 1
NCT00003434	Terminated	Autologous DC pulsed with a mutated CEA epitope-CAP-1	NO	Induce CAP-1-specific antitumour T cell reaction	Phase 1
NCT00669734	Active, not recruiting	Falimarev	Sargramostim	A cancer vaccine comprised of a recombinant fowlpox viral vector encoding CEA, MUC-1 to induce CEA- and MUC1-specific antitumour reactions	Phase 1
NCT02338752	Completed	MV (mixed vaccines)	Standard treatment	An intravenous intralipid suspension with 5 various vaccines, including DPT, typhoid, *Staphylococcus aureus*, and paratyphoid A and B	Phase 1 Phase 2
NCT03153410	Recruiting	GVAX	Cyclophosphamide, pembrolizumab, IMC-CS4	Pancreatic cell lines secreting GM-CSF to induce DC maturation	Early phase 1
NCT02648282	Recruiting	GVAX	Cyclophosphamide, pembrolizumab and radiation	Pancreatic cell lines secreting GM-CSF to induce DC maturation	Phase 2
NCT03161379	Recruiting	GVAX	Cyclophosphamide, nivolumab and radiation	Pancreatic cell lines secreting GM-CSF to induce DC maturation	Phase 2
NCT01896869	Suspended	GVAX	Ipilimumab, FOLFIRINOX	Pancreatic cell lines secreting GM-CSF to induce DC maturation	Phase 2
NCT00836407	Completed	GVAX	Ipilimumab	Pancreatic cell lines secreting GM-CSF to induce DC maturation	Phase 1
NCT01417000	Completed	GVAX and CRS-207	Cyclophosphamide	Pancreatic cell lines secreting GM-CSF to induce DC maturation in combination with a recombinant live-attenuated *Listeria monocytogenes* strain engineered to secrete mesothelin into the cytoplasm of infectious APCs	Phase 2
NCT03190265	Recruiting	GVAX and CRS-207	Cyclophosphamide, nivolumab, ipilimumab	Increase the mesothelin-specific antitumour activity and enhance the antigen-presenting ability of APCs	Phase 2
NCT02004262	Completed	GVAX and CRS-207	Cyclophosphamide	Increase the mesothelin-specific antitumour activity and enhance the antigen-presenting ability of APCs	Phase 2
NCT03006302	Recruiting	GVAX and CRS-207	Epacadostat, pembrolizumab, cyclophosphamide	Increase the mesothelin-specific antitumour activity and enhance the antigen-presenting ability of APCs	Phase 2
NCT02960594	Completed	INO-1400; INO-1401	INO-9012: a DNA molecule (plasmid) encoding IL-12	A synthetic telomerase reverse transcriptase (TERT) DNA vaccine to induce TERT-specific antitumour reaction	Phase 1
NCT00128622	Completed	Autologous dendritic cells infected with recombinant fowlpox-CEA(6D)-TRICOM vaccine	Denileukin diftitox	DCs were mixed with recombinant fowlpox-TRICOM to produce the vaccine and induce a CEA-specific antitumour reaction	Phase 1
NCT00529984	Completed	AVX701	NO	CEA(6D) VRP vaccine to induce a CEA-specific immune response targeting the mutated CEA epitope -CAP-1(6D)	Phase 1 Phase 2
NCT03552718	Recruiting	YE-NEO-001	NO	Recombinant yeast vaccine engineered to express multiple neoepitopes based on individual tumour molecular profiles	Phase 1
NCT00245362	Completed	CG 8020 and CG 2505	NO	Allogeneic cancer cell lines engineered to secrete GM-CSF to enhance the antigen-presenting ability of DCs	Phase 2
NCT00019084	Completed	Mutant p53 peptide- or ras peptide-pulsed APCs	GM-CSF, mutated peptide-stimulated autologous lymphocytes or TILs	APCs pulsed with synthetic version of the patient’s mutated p53 or ras peptide to induce a mutated peptide-specific antitumour reaction	Phase 2
NCT00002475	Completed	Allogeneic or autologous tumour cell vaccine	Recombinant IFN α, recombinant interferon γ, sargramostim, cyclophosphamide	Induce an adaptive immune reaction	Phase 2
NCT00027534	Completed	Autologous dendritic cells infected with recombinant fowlpox-CEA-TRICOM vaccine	NO	Increase the CEA-specific immune response	Phase 1
NCT00004604	Completed	CEA RNA-pulsed DC cancer vaccine	NO	Increase the CEA-specific immune response	Phase 1
NCT02151448	Active, not recruiting	Autologous alpha-DC1 loaded with autologous tumour material	Celecoxib, IFNα-2b, rintatolimod	Increase the adaptive antitumour response	Phase 1 Phase 2
NCT03300843	Recruiting	DC vaccine with defined immunogenic neoepitopes	NO	Induce a personalized mutated neoantigen immune response	Phase 2
NCT01088789	Recruiting	PANC 10.05 pcDNA-1/GM-Neo and PANC 6.03 pcDNA-1 neo vaccine	With or without cyclophosphamide	Increase the antitumour cellular response	Phase 2
NCT01595321	Active, not recruiting	PANC 10.05 pcDNA-1/GM-Neo and PANC 6.03 pcDNA-1/GM-Neo vaccine	Cyclophophamide, radiation, FOLFIRINOX	Increase the antitumour cellular response	Not applicable
NCT01342224	Completed	GV1001	Immune adjuvant, GM-CSF, gemcitabine	A telomerase vaccine	Phase 1

## Conclusion

In this review, we summarized the characteristics of the PDAC TME, including the cancer epithelial cell properties, the role of stromal cells and matrix in the immunosuppressive TME, the complex network among tumour-infiltrating immune cells and how these cells orchestrate the shape and programme of the PDAC TME. We have also covered the current and future aspects of immunotherapy for PDAC from various perspectives in this review. mAb-based immunotherapy still has the potential to enhance the treatment of PDAC. However, the absence of TAAs restricts its progression, and the strategy for improving the suboptimal selection of mAb-based therapy involves combinations with other approaches or exploration of TSAs, especially neoantigen-targeting mAbs, from TIBs [86], as the latter is emerging as a promising field. Vaccines may have dual roles in the treatment of PDAC. On the one hand, they can theoretically induce or enhance the naturally occurring antitumour response and improve the functions of transferred antitumour effector cells. However, they may have the adverse effect of inducing tumour-specific immune tolerance through Treg cells, which at least in part underlies the modest effect observed with vaccine treatment. For GVAX vaccines, GM-CSF alone might not be sufficient to induce APC maturation. Recent advances in isolating neoantigen-targeting antibodies from TIBs have given rise to a promising approach for both vaccine and mAb therapies as well as for selecting scFvs for CAR-T therapy. ACT with genetically engineered cells has achieved promising results in some solid tumours in preclinical studies but not in any clinical trials. ACT-based therapy must be immensely improved to exploit PDAC-targeting cells because PDAC has relatively few TAAs. Furthermore, the high stromal density and absence of angiogenesis dampen the infiltration of infused cells, and the suppressive TME also inactivates infiltrating cells. Promisingly, substantial progress has been made regarding PDAC TILs in recent years [135–137]. These results exploited potential tools to obtain multiple tumour-specific colonies and even a single TIL colony specific for endogenous tumour cells. The strategy of identifying and sequencing neoantigen-specific TCRs to engineer lymphocytes for ACT is expected, as Rosenberg and his team have made significant progress in this field [153–155].

## Data Availability

Not applicable.

## Abbreviations

ABC: ATP-binding cassette
ACT: Adoptive cell therapy
ACT: Adoptive cell therapy/adoptive cell transferring
ADCC: Antibody-dependent cellular cytotoxicity
APCs: Antigen-specific cells
Arg1: Arginase-1
CAFs: Cancer-associated fibroblasts
CAR: Chimeric antigen receptor
CEA: Carcinoembryonic antigen
CSCs: Cancer stem cells
CTLA-4: Cytotoxic T lymphocyte-associated antigen 4
Cy: Cyclophosphamide
DCs: Dendritic cells
DLL4: Delta-like ligand 4
EGFR: Epidermal growth factor receptor
EMT: Epithelial-to-mesenchymal transition
Gem: Gemcitabine
GM-CSF/CSF2: Granulocyte macrophage colony-stimulating factor
GVAX: GM-CSF-secreting tumour cells
HEV: High endothelial venules
ICI: Immune checkpoint inhibitor
ICI: Immune checkpoint inhibitor
IDO: Indoleamine 2,3-dioxygenase
iNOS: Inducible nitric oxide synthase
mAb: Monoclonal antibody
M-CSF/CSF1: Macrophage colony-stimulating factor
MDSCs: Myeloid-derived suppressive cells
MSLN: Mesothelin
OS: Overall survival
OVA: Ovalbumin
PanIN: Pancreatic intraepithelial neoplasia
PDAC: Pancreatic ductal adenocarcinoma
PD-L1: Programmed cell death protein ligand-1
PSCs: Pancreatic stellate cells
ROS: Reactive oxygen species
TAAs: Tumours associated antigens
TAMs: Tumour-associated macrophages
TANs: Tumour-associated neutrophils
Tconv: Conventional T cells
TGF-β: Transforming growth factor-β
TIBs: Tumour-infiltrating B cells
TILs: Tumour-infiltrating lymphocytes
TLS: Tertiary lymphoid structures
TME: Tumour microenvironment
Treg cells: Regulatory T cells
TSAs: Tissue-specific antigens
VEGF: Vascular endothelial growth factor
